# Corrigendum: Transcriptome Profiling of the Ovarian Cells at the Single-Cell Resolution in Adult Asian Seabass

**DOI:** 10.3389/fcell.2021.714482

**Published:** 2021-06-23

**Authors:** Xiaoli Liu, Wei Li, Yanping Yang, Kaili Chen, Yulin Li, Xinping Zhu, Hua Ye, Hongyan Xu

**Affiliations:** ^1^Key Laboratory of Freshwater Fish Reproduction and Development, Ministry of Education, Key Laboratory of Aquatic Sciences of Chongqing, College of Fisheries, Southwest University, Chongqing, China; ^2^Key Laboratory of Tropical & Subtropical Fishery Resource Application & Cultivation of Ministry of Agriculture and Rural Affairs, Pearl River Fisheries Research Institute, Chinese Academy of Fishery Sciences, Guangzhou, China

**Keywords:** single-cell, transcriptome, Asian seabass, ovary, aquatic animal

In the original article, we neglected to include the grant number for the funder Fundamental Research Funds for the Central Universities. The grant number is SWU020014 to Hongyan Xu. The corrected funding statement appears below.

**FUNDING**

“This research work was supported by grants from the National Natural Science Foundation of China (31873035), the Fundamental Research Funds for the Central Universities (SWU020014 to HX) (2021), Science and Technology Program of Guangzhou (201904010172), Social Public Welfare Research (2019HY-XKQ02), Open Project of State Key Laboratory of Freshwater Ecology and Biotechnology (2019FB01), Central Public-interest Scientific Institution Basal Research Fund, CAFS (2020TD35, 2020ZJTD01), Guangdong Agricultural Research System, Grant/Award Number (2019KJ150), China-ASEAN Maritime Cooperation Fund (CAMC-2018F), International Agricultural Exchange and Cooperation (2130114), Science and Technology Program of Guangdong Provincial, Grant/Award Number (2019B030316029), and National Freshwater Genetic Resource Center (NFGR-2020).”

In the published article, there was an error in affiliations for authors Kaili Chen, Yulin Li, and Xinping Zhu. Instead of “Kaili Chen^2^, Yulin Li^2^, Xinping Zhu^1^,” it should be “Xiaoli Liu^1,2^, Wei Li^2^, Yanping Yang^2^, Kaili Chen^1^, Yulin Li^1^, Xinping Zhu^2^, Hua Ye^1^ and Hongyan Xu^1,2^.”

The authors apologize for these errors and state that this does not change the scientific conclusions of the article in any way. The original article has been updated.

