# Supplementary figures and images for: Transcriptome Profiling of the Ovarian Cells at the Single-Cell Resolution in Adult Asian Seabass
Source: Front Cell Dev Biol. 2021 Mar 29;9:647892. doi: 10.3389/fcell.2021.647892 (PMC8039529; doi:10.3389/fcell.2021.647892)

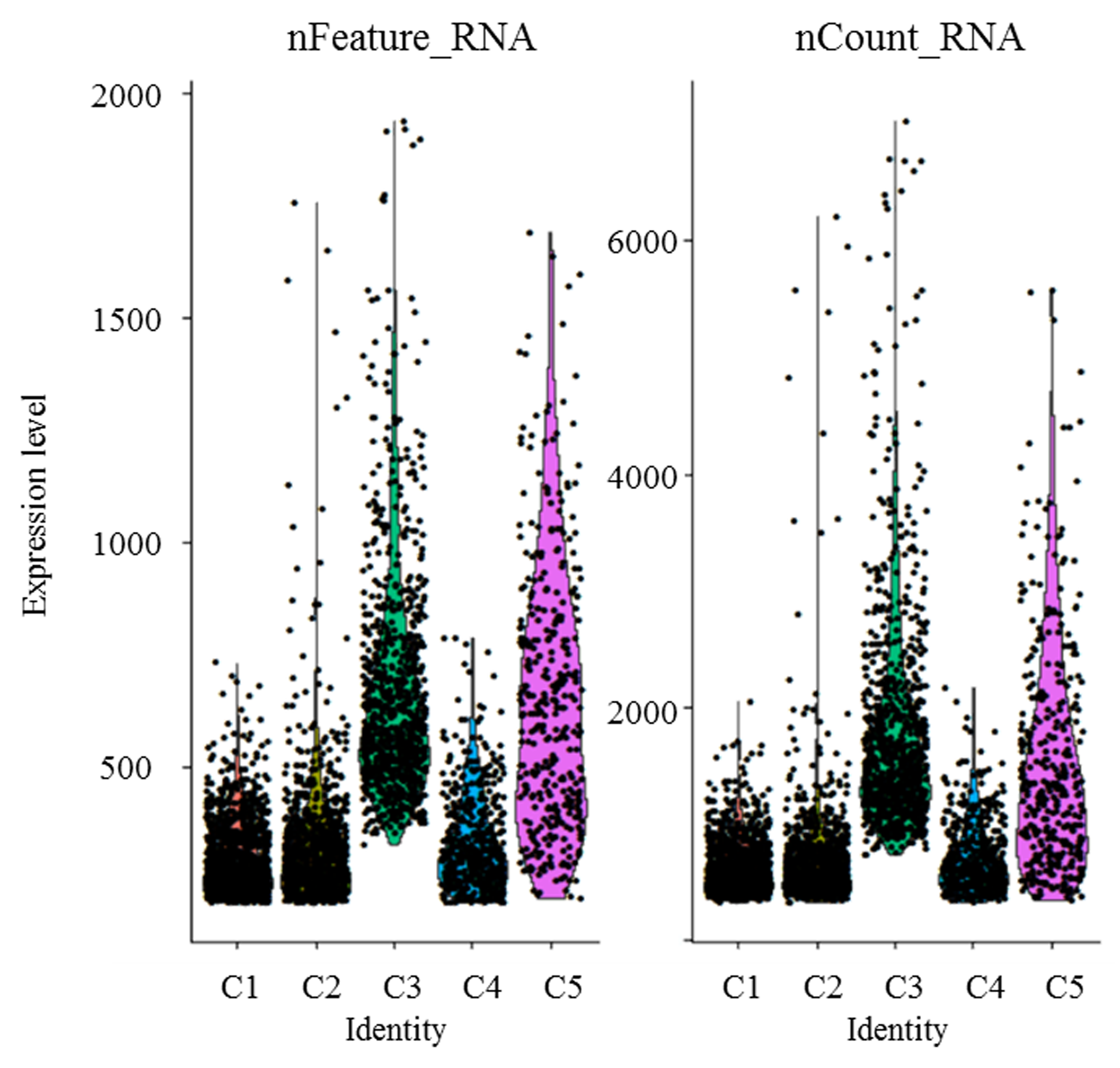

Supplement: Supplementary Figure 1 — Violin plots of 5 cell clusters. [file Image_1.TIF]

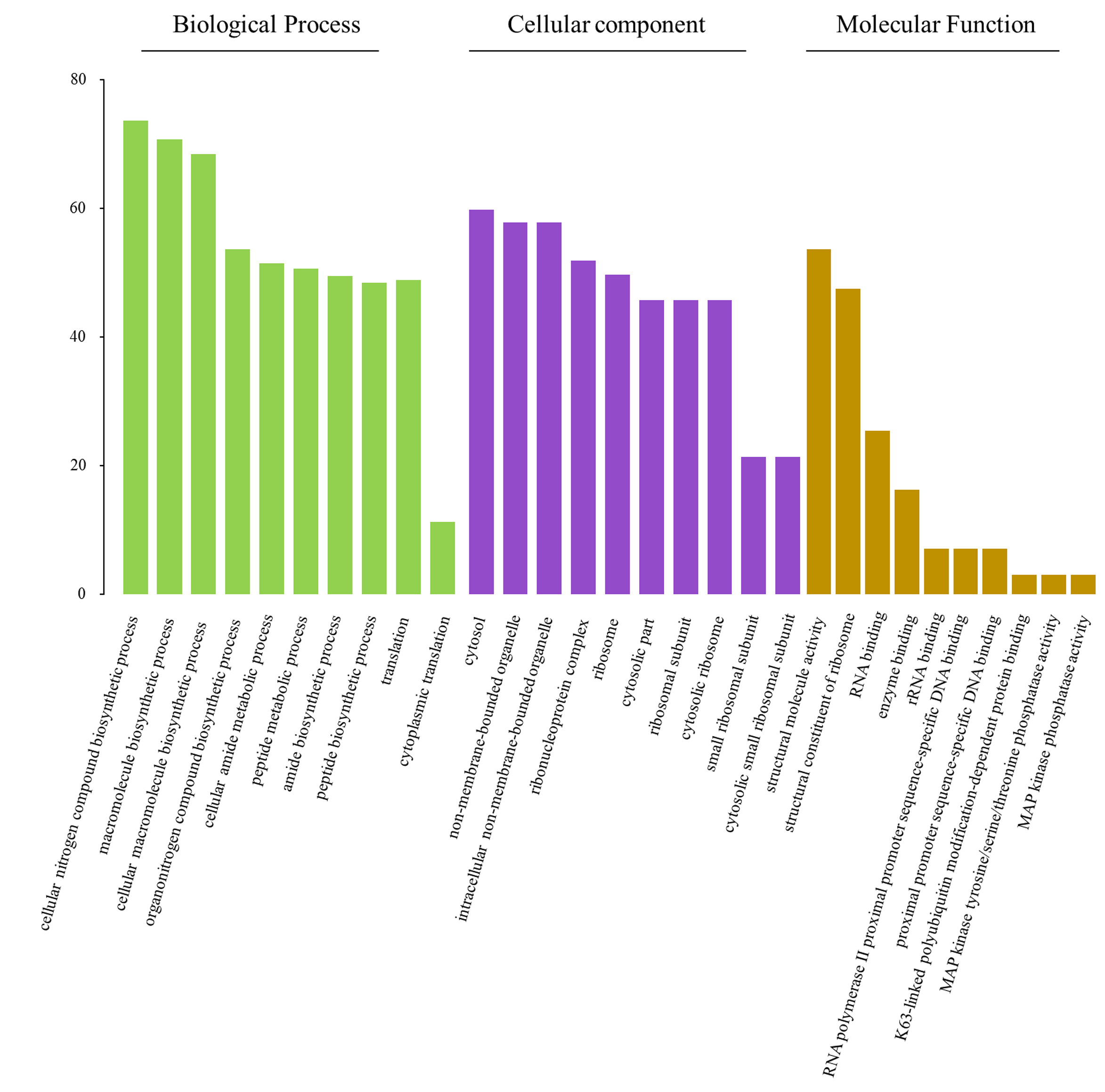

Supplement: Supplementary Figure 2 — Significantly enriched GO terms of DEGs in 5 clusters. [file Image_2.TIF]

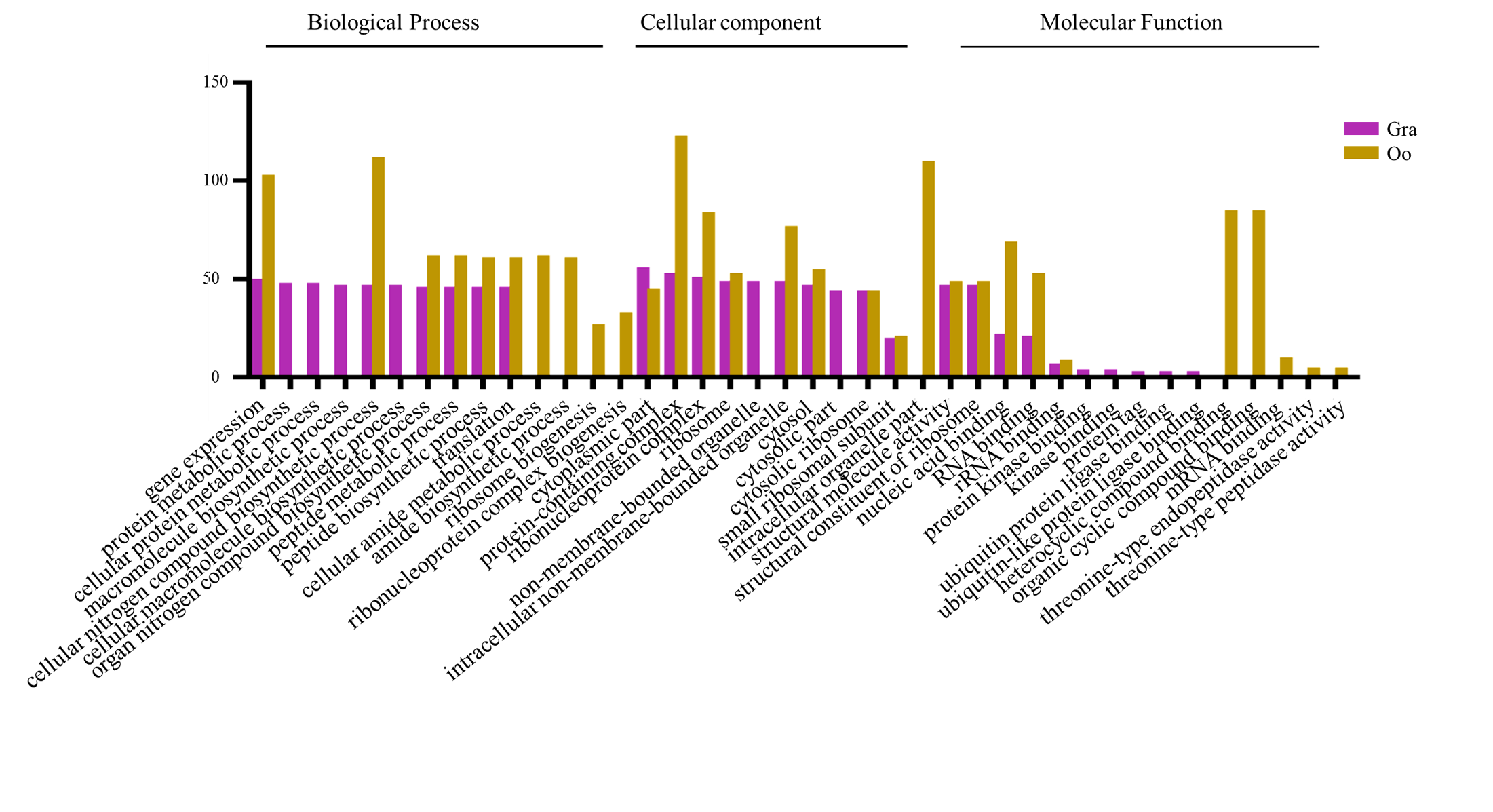

Supplement: Supplementary Figure 3 — Significantly enriched GO terms of zar1 transcripts enriched cells (zar1+) and dnajb1 transcripts enriched cells (dnajb1+). Oo and Gra represent zar1 transcripts enriched germ cells (zar1+) and dnajb1 transcripts enriched granulosa cells, respectively. [file Image_3.TIF]
