# Supplementary material for: Transcriptome Profiling of the Ovarian Cells at the Single-Cell Resolution in Adult Asian Seabass
Source: Front Cell Dev Biol. 2021 Mar 29;9:647892. doi: 10.3389/fcell.2021.647892 (PMC8039529; doi:10.3389/fcell.2021.647892)
Supplement: Supplementary file 4 [file Table_1.DOCX]

**Transcriptome profiling the ovarian cells in adult Asian seabass**

Xiaoli Liu^b^,Wei Li^b^, Yanping Yang^b^, Kaili Chen^a,c^, Yulin Li^a,c^, Xinping Zhu^b^ , Hua Ye^a,c^, Hongyan Xu^a,c*^

^a^Key Laboratory of Freshwater Fish Reproduction and Development (Ministry of Education), College of Fisheries, Southwest University, Chongqing, 402460, China

^b^Key Laboratory of Tropical & Subtropical Fishery Resource Application & Cultivation of Ministry of Agriculture and Rural Affairs, Pearl River Fisheries Research Institute, Chinese Academy of Fishery Sciences, Guangzhou, 510380

^c^ Key Laboratory of Aquatic Science of Chongqing 400175, China

**Table S1 Sequences of primers used for this study.**

| primer | Sequence (5’-3’) |
| --- | --- |
| *dnd1* probe N | CTACTGAAGGTGCTGCGTGTGC |
| *dnd1* probe T7C | TAATACGACTCACTATAGGGAGGAGCAGGGAGGGAAAGGGT |
| *nanos3* probe N | TGGTTTGGGGGCTCTTTCAT |
| *nanos3* probe T7C | TAATACGACTCACTATAGGGCGTCTTAGTGGTCTTCTTGCGGT |
| *zar1* probe N | AAAGGAGGCAGGAGCCCGAGA |
| *zar1* probe T7C | TAATACGACTCACTATAGGGGCCCTGAACGCACCACACATA |
| *hsd17b1* probe N | CTGGCGAAGAAGGAGCGTC |
| *hsd17b1* probe T7C | TAATACGACTCACTATAGGGTGTGGGTATCGACCTGTTGGA |
| *dnajb1* probe N | GCTGGAATGTTTGTGGGACTCT |
| *dnajb1* probe T7C | TAATACGACTCACTATAGGGTGGACACCGACGACCCTTTTG |
